# Supplementary material for: A Likelihood Approach for Real-Time Calibration of Stochastic Compartmental Epidemic Models
Source: PLoS Comput Biol. 2017 Jan 17;13(1):e1005257. doi: 10.1371/journal.pcbi.1005257 (PMC5240920; doi:10.1371/journal.pcbi.1005257)
Supplement: S1 File — (TAR.GZ) [file pcbi.1005257.s014.tar.gz › HSPH_Online-SI-Revision/EnKF07/n10-extreme/n10-extreme_EnKF_table0.pdf]

1	R0	{2.68692, 12.5884}	{2.00912, 7.87983}	{{0.117376, 5.06852}, {3., 12.5395}}	{38.2762, 37.6225}	inf	2477.	437.454	{56.0334, 852.407}	84.7468	1.05172	newly	1264.	305.37	{43., 444.}	82.6907	0.906539	Reff	0.771415	0.449905	{0.0256711, 0.665533}	43.3094	ar	9089.	18418.2	{17172., 20157.}	102.642
2	R0	{1.79802, 12.5412}	{2.73419, 11.5059}	{{2.24926, 6.61731}, {3., 14.5332}}	{52.0669, 27.6441}	inf	1208.	469.446	{99.7799, 364.94}	94.192	0.788852	newly	678.	272.15	{46., 229.}	93.2109	0.788691	Reff	1.00509	0.664458	{0.225464, 1.20797}	50.5211	ar	7412.	17037.9	{15518., 17440.}	129.869
3	R0	{2.6174, 10.1105}	{2.22565, 5.11407}	{{1.57719, 3.78189}, {3., 4.18466}}	{28.1228, 63.9292}	inf	2244.	884.479	{61.5876, 3106.69}	85.3046	0.875623	newly	1325.	1291.45	{60., 5481.}	125.545	0.752735	Reff	0.662726	0.531261	{0.151296, 1.36508}	56.5304	ar	9109.	20065.6	{17307., 30447.}	120.283
4	R0	{2.27532, 14.3149}	{2.70785, 12.327}	{{2.10031, 6.68539}, {3., 16.4613}}	{21.758, 30.8833}	inf	1860.	581.993	{123.887, 426.13}	97.3929	0.993614	newly	862.	304.	{47., 192.}	95.942	0.933973	Reff	0.820025	0.724825	{0.270813, 1.38709}	48.4492	ar	8478.	17306.8	{15360., 18524.}	104.138
5	R0	{2.96649, 18.0674}	{2.62326, 8.00664}	{{1.9514, 1.11706}, {3., 17.4254}}	{12.858, 55.8846}	inf	2876.	437.755	{34.1839, 931.173}	84.9002	1.12816	newly	1043.	1287.33	{51., 6361.}	154.582	0.857256	Reff	0.663011	0.680242	{0.221002, 1.9512}	57.3797	ar	9476.	18425.6	{16417., 26061.}	94.4447
6	R0	{2.94778, 16.0002}	{2.0406, 8.04088}	{{0.196524, 5.07087}, {3., 12.5864}}	{32.7945, 49.7452}	inf	2859.	409.381	{65.3052, 734.235}	87.3599	1.1537	newly	1175.	290.93	{50., 379.}	85.2111	0.93084	Reff	0.814766	0.455213	{0.0426682, 0.679806}	45.2793	ar	9321.	18188.6	{17040., 19995.}	95.1358
7	R0	{2.17562, 11.7184}	{2.39694, 4.95705}	{{1.54183, 1.81991}, {3., 8.40986}}	{31.2861, 57.6986}	inf	1758.	410.904	{28.7213, 1052.92}	91.4615	1.18123	newly	1027.	784.94	{37., 2842.}	132.325	1.02335	Reff	0.998827	0.554359	{0.151798, 1.08447}	61.0612	ar	8345.	19067.	{17303., 24582.}	128.485
8	R0	{2.7904, 17.8966}	{2.61469, 6.06826}	{{1.57939, 3.63075}, {3., 8.50576}}	{16.3621, 66.0927}	inf	2748.	497.467	{163.248, 577.227}	87.9315	0.963834	newly	978.	574.11	{148., 673.}	89.9499	0.585302	Reff	0.871163	0.554632	{0.108216, 0.78112}	53.0828	ar	9285.	17675.5	{15914., 25494.}	90.3661
9	R0	{2.51574, 16.376}	{2.59463, 6.1849}	{{1.55546, 3.63208}, {3., 8.54135}}	{22.273, 62.2319}	inf	2361.	428.815	{181.669, 571.031}	89.2571	0.959548	newly	932.	542.47	{131., 690.}	92.7221	0.616088	Reff	0.84076	0.594133	{0.151737, 0.817235}	49.0678	ar	8937.	17579.	{15796., 23632.}	96.6987
10	R0	{2.56405, 18.1787}	{2.62805, 6.21566}	{{1.60081, 3.62564}, {3., 8.70411}}	{20.287, 65.808}	inf	2387.	445.532	{229.558, 454.394}	88.6177	0.93284	newly	979.	568.55	{130., 671.}	89.8355	0.604468	Reff	0.996903	0.618174	{0.130957, 0.939643}	50.4016	ar	9005.	17835.2	{15927., 24038.}	98.0589
11	R0	{2.475, 17.1709}	{2.54585, 3.81915}	{{0.615019, 3.16788}, {3., 4.8196}}	{24.7133, 77.758}	inf	2191.	654.998	{89.052, 1540.27}	81.968	0.894539	newly	841.	935.26	{99., 2545.}	108.145	0.523967	Reff	0.935303	0.669524	{0.141201, 1.47461}	60.0968	ar	8870.	17640.	{15344., 21470.}	98.8731
12	R0	{2.14974, 11.8619}	{2.62065, 6.26733}	{{1.62141, 3.60942}, {3., 8.82101}}	{33.1756, 47.1642}	inf	1788.	311.304	{213.864, 444.012}	84.0021	0.829877	newly	1021.	356.11	{121., 586.}	73.3604	0.620948	Reff	1.0409	0.590398	{0.118717, 0.942602}	55.0787	ar	8414.	17693.5	{15929., 24051.}	110.286
13	R0	{2.20385, 14.4767}	{2.18522, 3.35425}	{{0.769505, 2.15325}, {3., 4.55876}}	{46.3636, 76.83}	inf	1745.	470.097	{99.1538, 558.593}	87.0371	0.919972	newly	848.	560.45	{102., 479.}	90.8573	0.602921	Reff	0.831733	0.442754	{0.138445, 0.644343}	54.5676	ar	8347.	16953.7	{15740., 19074.}	103.111
14	R0	{2.35671, 13.8357}	{2.59824, 8.13063}	{{1.96801, 1.17575}, {3., 17.6373}}	{23.442, 62.6019}	inf	2049.	519.454	{67.9306, 769.494}	83.1782	0.921499	newly	946.	1416.8	{36., 7710.}	171.342	0.790155	Reff	0.81118	0.692062	{0.108816, 1.96781}	58.1363	ar	8719.	18771.7	{16689., 26163.}	115.297
15	R0	{1.91368, 10.1082}	{2.72798, 12.1179}	{{2.12597, 6.70549}, {3., 15.931}}	{42.5516, 47.4857}	inf	1215.	544.346	{103.588, 414.111}	97.8444	0.778932	newly	838.	294.25	{38., 203.}	93.4666	0.862314	Reff	0.888904	0.737335	{0.235014, 1.41984}	51.009	ar	7486.	17399.7	{15470., 18669.}	132.43
16	R0	{2.48727, 14.882}	{2.63546, 7.74995}	{{1.98419, 1.19703}, {3., 17.3779}}	{19.7118, 60.0941}	inf	2351.	471.977	{27.2782, 1307.98}	83.9397	1.04516	newly	1082.	1274.86	{30., 6207.}	149.203	0.842497	Reff	0.913574	0.678944	{0.137951, 1.98399}	58.1439	ar	8894.	18331.4	{16338., 27357.}	106.11
17	R0	{2.46795, 13.1915}	{2.38132, 5.18312}	{{1.37383, 1.97588}, {3., 9.13749}}	{25.4996, 60.7087}	inf	2177.	385.112	{14.8936, 355.375}	91.1978	1.31943	newly	1116.	698.37	{10., 557.}	121.901	1.1011	Reff	0.942263	0.513589	{0.0853265, 0.976097}	64.723	ar	8796.	18818.4	{17172., 23016.}	113.943
18	R0	{2.96603, 16.7543}	{2.62495, 7.63955}	{{1.95346, 1.12089}, {3., 17.2184}}	{12.828, 56.1789}	inf	2830.	422.935	{38.8093, 927.375}	85.3387	1.13368	newly	1119.	1214.89	{46., 2854.}	142.164	0.845022	Reff	0.741508	0.697972	{0.2262, 1.95326}	54.2373	ar	9394.	18386.1	{16504., 25267.}	95.722
19	R0	{2.25216, 9.90465}	{2.36331, 4.89256}	{{1.34226, 1.96456}, {3., 8.82778}}	{31.8656, 50.6034}	inf	1991.	516.652	{132.988, 1324.52}	84.7217	0.863039	newly	1350.	915.21	{88., 4765.}	105.333	0.743163	Reff	0.932845	0.539871	{0.0512767, 1.31346}	57.0819	ar	8631.	19168.4	{17405., 25570.}	122.087
20	R0	{2.70837, 18.1923}	{2.62166, 6.14398}	{{1.57266, 3.62926}, {3., 8.65871}}	{17.6302, 66.2276}	inf	2671.	388.669	{193.99, 453.223}	90.1523	0.992859	newly	986.	477.9	{132., 639.}	83.8053	0.605787	Reff	0.903241	0.576623	{0.126844, 0.795669}	54.5718	ar	9183.	17621.6	{15890., 24086.}	91.8937
21	R0	{2.30288, 15.5877}	{2.41465, 1.90808}	{{1.61198, 0.740494}, {3., 3.31143}}	{29.0509, 87.7591}	inf	2049.	591.419	{312.44, 799.877}	71.8642	0.587442	newly	836.	1103.97	{415., 1645.}	59.5347	0.199232	Reff	0.842854	0.627419	{0.254601, 1.1308}	46.3884	ar	8674.	17068.8	{15793., 19093.}	96.7813
22	R0	{2.82208, 10.7544}	{2.24991, 4.75144}	{{1.59741, 3.5151}, {3., 6.77011}}	{26.5794, 55.8186}	inf	2783.	370.84	{86.6695, 638.6}	88.8619	1.18294	newly	1622.	463.01	{59., 756.}	91.881	0.99527	Reff	0.73233	0.36115	{0.045808, 0.690351}	63.4408	ar	9333.	18734.1	{17083., 22418.}	100.729
23	R0	{1.69923, 10.1366}	{2.33196, 6.64622}	{{0.585367, 0.486626}, {3., 10.9302}}	{68.2114, 43.5659}	inf	1080.	605.025	{34.0672, 1157.66}	105.412	0.881686	newly	667.	861.62	{23., 1986.}	147.253	0.806791	Reff	0.906029	0.606173	{0.10431, 1.05634}	50.0258	ar	7055.	17303.9	{15413., 20323.}	145.272
24	R0	{2.64944, 19.054}	{2.41335, 1.98172}	{{1.60611, 0.816482}, {3., 3.32922}}	{23.9948, 89.5994}	inf	2322.	551.412	{307.201, 759.269}	76.2527	0.665109	newly	778.	1102.93	{406., 2027.}	74.0758	0.232724	Reff	0.697333	0.619734	{0.134646, 1.07983}	53.5293	ar	9147.	17060.2	{15692., 18160.}	86.5112
25	R0	{2.53191, 10.9214}	{2.55632, 6.30242}	{{1.56713, 3.63987}, {3., 8.48087}}	{21.2936, 42.2929}	inf	2411.	308.196	{169.992, 608.842}	87.2171	0.959446	newly	1327.	369.7	{139., 832.}	75.5388	0.722487	Reff	0.740077	0.637774	{0.193372, 0.926539}	45.1585	ar	9009.	17438.3	{15729., 23712.}	93.5649
26	R0	{1.89155, 6.76944}	{2.34117, 5.09692}	{{1.2157, 1.94105}, {3., 9.10696}}	{42.9422, 52.8509}	inf	989.	373.137	{17.8293, 413.266}	96.4119	1.02875	newly	913.	685.16	{22., 1180.}	130.565	1.05569	Reff	0.863871	0.491409	{0.123925, 0.862411}	65.6493	ar	7348.	18830.3	{17257., 21761.}	156.264
27	R0	{2.63149, 15.6979}	{2.63488, 7.81966}	{{1.98597, 1.15481}, {3., 17.6227}}	{16.8096, 58.6713}	inf	2448.	481.911	{19.8727, 1426.39}	83.7569	1.07927	newly	1086.	1288.38	{27., 5958.}	147.011	0.837497	Reff	0.945758	0.699665	{0.195037, 1.98577}	56.9109	ar	9104.	18265.7	{16181., 27398.}	100.633
28	R0	{2.85141, 12.6992}	{2.24619, 5.2102}	{{1.59457, 3.91311}, {3., 4.21663}}	{26.2279, 67.253}	inf	2712.	863.212	{90.2012, 2781.75}	82.778	0.946573	newly	1362.	1215.6	{87., 3628.}	119.924	0.775269	Reff	0.727965	0.519698	{0.159031, 1.17008}	55.6081	ar	9317.	19847.1	{17273., 27042.}	113.02
29	R0	{2.54244, 12.9085}	{2.30781, 6.7769}	{{0.506568, 0.55756}, {3., 11.4736}}	{31.5588, 47.5005}	inf	2318.	484.675	{68.0323, 412.068}	91.3895	1.13154	newly	1154.	658.35	{44., 1356.}	95.1187	0.878668	Reff	0.769342	0.581008	{0.0974011, 1.01177}	48.9738	ar	9034.	17131.2	{15456., 20080.}	89.6305
30	R0	{2.10982, 11.7636}	{2.3449, 6.71559}	{{0.548335, 0.502725}, {3., 11.6006}}	{43.6213, 42.9121}	inf	1626.	635.198	{66.9433, 1214.24}	95.6925	0.953202	newly	882.	921.55	{36., 2259.}	125.978	0.80088	Reff	0.892032	0.587108	{0.105654, 1.11712}	52.0772	ar	8224.	17433.7	{15485., 22766.}	111.986
31	R0	{2.76312, 15.5396}	{2.11449, 8.31131}	{{0.176303, 5.04216}, {3., 12.8285}}	{33.5905, 46.5153}	inf	2551.	381.766	{61.8998, 699.566}	87.0648	1.13446	newly	1066.	250.82	{40., 347.}	85.2664	0.929035	Reff	0.809318	0.460555	{0.0374778, 0.669108}	44.5517	ar	9220.	18264.8	{17278., 18973.}	98.0997
32	R0	{2.21727, 15.5439}	{2.62886, 3.85806}	{{1.93759, 2.59882}, {3., 5.45172}}	{26.0104, 75.1796}	inf	1810.	828.551	{132.923, 2382.56}	80.5387	0.675815	newly	784.	1211.35	{133., 2934.}	125.239	0.428941	Reff	0.888682	0.677104	{0.224894, 1.64623}	60.3515	ar	8359.	18198.8	{15152., 22900.}	117.714
33	R0	{2.72912, 10.8159}	{2.03829, 7.56055}	{{0.0988035, 4.79645}, {3., 11.335}}	{37.0254, 33.4003}	inf	2166.	496.747	{132.894, 955.238}	80.8282	0.820043	newly	1179.	358.39	{105., 537.}	77.4852	0.689373	Reff	0.537091	0.459582	{0.0224461, 0.69944}	46.0517	ar	9196.	18806.6	{17623., 19839.}	104.508
34	R0	{2.20928, 10.1308}	{2.30716, 5.7619}	{{1.62965, 0.441221}, {3., 14.965}}	{29.8605, 56.0181}	inf	1753.	869.291	{119.216, 2267.05}	81.7415	0.718313	newly	1080.	1258.31	{109., 4815.}	133.549	0.655934	Reff	0.754911	0.590529	{0.168617, 1.62924}	59.8842	ar	8388.	19267.5	{16251., 29225.}	130.644
35	R0	{2.10103, 15.2673}	{2.35289, 6.89374}	{{0.549515, 0.522374}, {3., 11.1444}}	{44.4627, 54.8464}	inf	1737.	497.804	{45.034, 1070.35}	95.0837	1.09183	newly	782.	755.02	{30., 1357.}	125.535	0.863359	Reff	0.894198	0.58807	{0.100511, 1.08707}	51.7471	ar	8264.	17326.6	{15554., 19632.}	109.663
36	R0	{2.74914, 19.2775}	{2.4487, 1.88981}	{{1.67411, 0.732964}, {3., 3.30949}}	{21.5136, 90.1968}	inf	2701.	607.126	{261.712, 754.853}	78.7379	0.730933	newly	946.	1108.39	{325., 1914.}	54.8742	0.21575	Reff	0.859106	0.621159	{0.324847, 1.03653}	45.4697	ar	9301.	16956.6	{15619., 18814.}	82.3099
37	R0	{2.78161, 16.0136}	{2.548, 6.00933}	{{1.46026, 3.56936}, {3., 8.4493}}	{18.5662, 62.4736}	inf	2648.	400.378	{125.569, 641.931}	89.7731	1.01706	newly	1097.	491.55	{130., 843.}	83.7548	0.673221	Reff	0.778294	0.569201	{0.106699, 0.798513}	42.995	ar	9240.	17452.2	{15839., 23461.}	88.8763
38	R0	{2.4454, 8.33501}	{2.32713, 4.6149}	{{1.09045, 1.89114}, {3., 8.39593}}	{29.2535, 45.0502}	inf	1846.	430.794	{78.2194, 987.549}	86.2562	0.984865	newly	1286.	781.41	{54., 1079.}	112.233	0.877944	Reff	0.652922	0.475911	{0.0892512, 0.83034}	56.6655	ar	8853.	18901.4	{17255., 20132.}	113.503
39	R0	{2.9154, 12.3861}	{2.15962, 4.97867}	{{0.83617, 1.85443}, {3., 8.68073}}	{28.7095, 59.8044}	inf	2561.	282.396	{47.7243, 225.552}	91.9731	1.31418	newly	1272.	333.18	{38., 373.}	94.3868	1.06757	Reff	0.603196	0.382715	{0.0969194, 0.485308}	57.0698	ar	9371.	18320.6	{17271., 19502.}	95.5031
40	R0	{2.40923, 15.1963}	{2.61267, 6.28914}	{{1.56948, 3.62779}, {3., 8.55621}}	{24.4479, 58.614}	inf	2019.	450.25	{220.393, 603.724}	87.3985	0.861787	newly	887.	592.6	{131., 891.}	90.8703	0.571996	Reff	0.893824	0.624459	{0.121622, 0.947209}	50.994	ar	8735.	17754.1	{15751., 23789.}	103.253
41	R0	{2.63137, 15.238}	{2.365, 6.67744}	{{0.512283, 0.608311}, {3., 11.2577}}	{28.6147, 56.179}	inf	2300.	514.895	{42.2847, 1117.48}	92.1199	1.19615	newly	964.	831.17	{24., 1886.}	117.874	0.92829	Reff	0.798621	0.578206	{0.103998, 1.01626}	49.9395	ar	9150.	17228.1	{15459., 19721.}	88.2852
42	R0	{2.48769, 19.4593}	{2.63891, 2.32396}	{{1.881, 0.356066}, {3., 3.53011}}	{19.5933, 88.0574}	inf	2205.	1045.97	{89.0607, 3325.11}	82.8784	0.756147	newly	727.	2443.74	{133., 6861.}	290.116	0.570059	Reff	0.77392	0.931759	{0.286555, 2.50432}	60.8701	ar	8932.	18560.7	{15251., 26484.}	107.8
43	R0	{2.61199, 10.214}	{2.24567, 4.90964}	{{1.61316, 3.78106}, {3., 6.91447}}	{28.5824, 51.9323}	inf	2530.	308.558	{59.8842, 430.77}	90.5548	1.22504	newly	1603.	370.8	{45., 471.}	91.3075	1.07002	Reff	0.846024	0.342768	{0.0402519, 0.67636}	65.9176	ar	9061.	18797.4	{17235., 23149.}	107.453
44	R0	{2.50994, 18.3382}	{2.60068, 4.99078}	{{2.22646, 3.82768}, {3., 5.73382}}	{21.7671, 72.7848}	inf	2193.	412.158	{180.577, 699.429}	83.6675	0.905387	newly	810.	677.25	{178., 1301.}	93.5988	0.52757	Reff	0.937463	0.735248	{0.152935, 2.08439}	62.6918	ar	8827.	17176.8	{15079., 22026.}	94.5942
45	R0	{2.44283, 16.8338}	{2.3461, 6.89874}	{{0.549645, 0.550781}, {3., 11.3355}}	{33.6108, 59.0185}	inf	2140.	538.378	{33.3486, 698.88}	93.6279	1.15314	newly	873.	799.1	{24., 1459.}	115.915	0.863758	Reff	0.849616	0.594524	{0.106677, 1.0266}	48.7878	ar	8878.	17406.2	{15544., 21805.}	96.0604
46	R0	{2.51815, 18.1462}	{2.47037, 1.92786}	{{1.7066, 0.71738}, {3., 3.30672}}	{24.4767, 89.376}	inf	2349.	474.33	{172.612, 639.924}	79.8071	0.789133	newly	861.	844.01	{207., 1640.}	47.4413	0.264807	Reff	0.909556	0.59676	{0.325974, 0.933072}	50.351	ar	8978.	16780.4	{15549., 18204.}	86.9061
47	R0	{2.88885, 16.8987}	{2.35551, 6.86302}	{{0.530286, 0.461549}, {3., 11.6971}}	{23.4639, 59.3873}	inf	2783.	563.773	{64.1078, 1213.09}	88.2837	1.13053	newly	1104.	866.32	{42., 2110.}	107.973	0.843638	Reff	0.890344	0.631627	{0.102832, 1.15394}	51.0987	ar	9337.	17486.	{15500., 22211.}	87.2762
48	R0	{2.58709, 13.8786}	{2.03473, 8.0465}	{{0.17754, 5.17838}, {3., 12.797}}	{39.2262, 42.0222}	inf	2523.	433.539	{84.9524, 775.175}	85.4722	1.05009	newly	1180.	306.61	{61., 400.}	83.5754	0.87582	Reff	0.854775	0.460018	{0.0399731, 0.701958}	46.91	ar	9146.	18060.8	{16888., 19902.}	97.4716
49	R0	{2.42959, 10.7132}	{2.36964, 4.75779}	{{1.36303, 1.955}, {3., 8.58566}}	{27.8235, 55.5895}	inf	2209.	507.527	{130.849, 1354.49}	85.4335	0.911846	newly	1386.	920.95	{88., 4557.}	105.852	0.756013	Reff	0.872223	0.544779	{0.081498, 1.33413}	55.8526	ar	8863.	19248.1	{17413., 25890.}	117.173
50	R0	{2.60513, 13.2796}	{2.54799, 8.02489}	{{2.00034, 1.12924}, {3., 17.7468}}	{19.9696, 66.0859}	inf	2476.	433.004	{30.1597, 1561.6}	83.0616	1.11558	newly	1231.	1209.	{38., 2864.}	134.944	0.913384	Reff	0.977184	0.719429	{0.192949, 2.00014}	54.7875	ar	9095.	18242.6	{16290., 24285.}	100.578
